# Contactless robotic system for linear catheter advancement using magnetic actuation

**DOI:** 10.1007/s11548-026-03751-8

**Published:** 2026-07-03

**Authors:** Lucas Primo Sianca, Jan Stallkamp, Javier Moviglia

**Affiliations:** Mannheim Institute for Intelligent Systems in Medicine, Heidelberg University, Theodor-Kutzer-Ufer 1–3, 68167 Mannheim, Baden-Württemberg Germany

**Keywords:** Robotic catheters, Magnetic actuation, Disposable cartridge, Sterile interface, Catheter navigation, Endovascular interventions

## Abstract

****Purpose**:**

Sterility management remains a challenge in robotic catheter manipulation. This work presents FlyCaD, an early-stage robotic catheter-drive prototype that transmits torque magnetically across a sealed sterile barrier and uses a disposable cartridge as the only component in direct contact with the catheter.

****Methods**:**

The system consists of a reusable static base module and a dynamic upper module carrying the disposable cartridge. Magnetic coupling enables catheter gripping, release, and guidewire rotation without mechanical contact across the sterile barrier. Linear motion is obtained through a clamp–translate–release–retract sequence. The prototype was evaluated in transparent bifurcated vascular phantoms using insertion–retraction cycles, combined guidewire–catheter manipulation, and branch-selection trials.

****Results**:**

The prototype completed 10/10 catheter insertion–retraction cycles and 10/10 combined guidewire–catheter cycles without loss of grip. Branch selection was successful on the first attempt in 8/10 trials. Commercial 4F–8F catheters were tested without hardware changes. The cartridge was easy to exchange and maintained magnetic torque transmission during operation. The levitation mechanism enabled contactless translation but lost stability above an unmeasured axial load threshold.

****Conclusion**:**

FlyCaD demonstrates the feasibility of contactless magnetic torque transmission across a sealed sterile wall for robotic catheter manipulation. The prototype is a functional proof of concept rather than a clinically validated platform. Future work will focus on instrumented characterization, improved linear actuation, wireless operation, and real-time control.

**Supplementary Information:**

The online version contains supplementary material available at https://doi.org/10.1007/s11548-026-03751-8.

## Introduction

Cardiovascular diseases remain a leading cause of death worldwide, and catheter-based interventions play a central role in their treatment [1, 2]. Catheters are used for vessel recanalization with stents or thrombolysis, aneurysm coiling, and transcatheter aortic valve replacement (TAVI) [3]. However, catheter navigation requires substantial operator expertise, as uncontrolled manipulation may lead to complications such as perforation, dissection, or vessel spasm. In addition, interventionalists are exposed to ionizing radiation, heavy lead aprons, and physically demanding working conditions during prolonged procedures. These limitations have motivated the development of robotic catheter systems to improve operator safety, reduce strain, and enable more ergonomic operation.

Despite these advantages, robotic catheter platforms have not yet become routine in many endovascular procedures. Earlier systems have been limited by high cost, infrastructure requirements, restricted catheter compatibility, limited reach, and the challenge of satisfying strict sterility requirements [4, 5]. Sterility is particularly important because robotic tools often contain multipart mechanical interfaces that are difficult to clean and sterilize, while single-use components increase procedural cost and environmental impact [6–8].

The main contribution of this work is a catheter-driving architecture in which torque is transmitted magnetically across a sealed sterile wall, reducing the catheter-contacting component to a disposable cartridge. The proposed system, FlyCaD (Flying Catheter Drive), combines contactless magnetic actuation with a compact sterile interface while preserving compatibility with commercial catheters. This study presents the prototype and evaluates its functional performance in bifurcated vascular phantoms.

## Materials and methods

### System overview

**Fig. 1:**
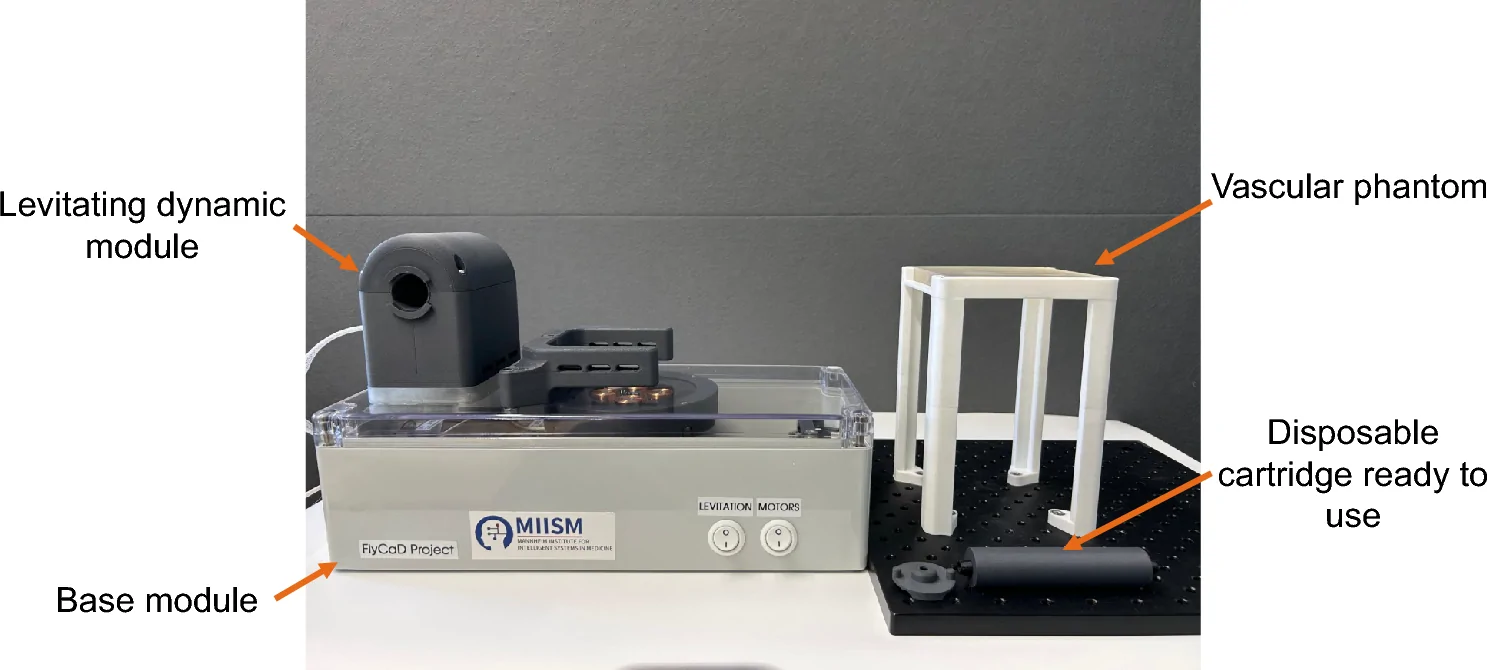
Frontal view of the FlyCaD prototype showing the main components in a stationary state, without actuation

The FlyCaD prototype is illustrated in Fig. 1. The system consists of a reusable static base module and a dynamic upper module responsible for catheter manipulation. The dynamic module carries a disposable cartridge, which forms the sterile catheter-contacting interface and can be replaced after use. The base module contains the actuation and control components. The prototype was evaluated in transparent vascular phantoms under simulated endovascular conditions.

### Magnetic actuation module

**Fig. 2:**
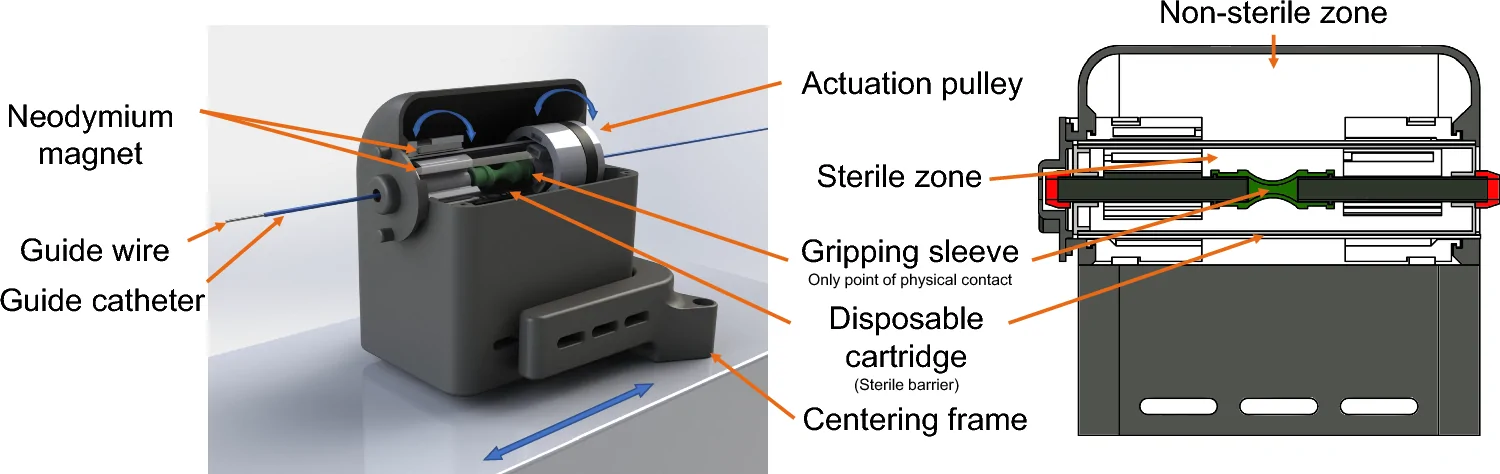
Actuation principle of the disposable cartridge. The cross-sectional schematic identifies the sterile zone, non-sterile zone, and sterile barrier formed by the sealed cartridge wall. The external pulley transmits torque magnetically to the internal cartridge components, while the gripping sleeve secures the catheter

The magnetic actuation module uses three permanent magnets inside the sealed disposable cartridge, magnetically coupled to three matching magnets embedded in an external drive pulley. Opposite poles face each other, generating an attractive magnetic coupling that transmits torque across the sterile barrier without mechanical contact. When the external pulley rotates, the internal magnets co-rotate accordingly. The internal magnets are attached to a thin tube that houses the catheter; this tube and the gripping sleeve are the only components in physical contact with the catheter (Fig. 2).

The design consists of two coupled pulley–magnet assemblies acting on a split tube. A central flexible partition allows relative motion between the two halves. When the pulleys rotate in opposite directions, the split section constricts a silicone sleeve, which grips the catheter. Reversing the motor commands releases the sleeve, allowing catheter advancement or repositioning. The catheter can therefore be gripped, released, and rotated bidirectionally under sterile separation. The silicone sleeve adapts to the catheter outer diameter through controlled radial constriction, allowing compatibility with off-the-shelf catheters within the tested size range.

### Linear advancement method

Linear catheter advancement is achieved by translating the dynamic module along a guided path. During operation, the system alternates between gripping and releasing the catheter. The sleeve first constricts around the catheter; the dynamic module then translates forward, advancing the catheter. The sleeve is subsequently released, and the dynamic module retracts without pulling the catheter back. Repetition of this clamp–translate–release–retract sequence enables incremental catheter advancement beyond the single-stroke travel of the module.

**Fig. 3:**
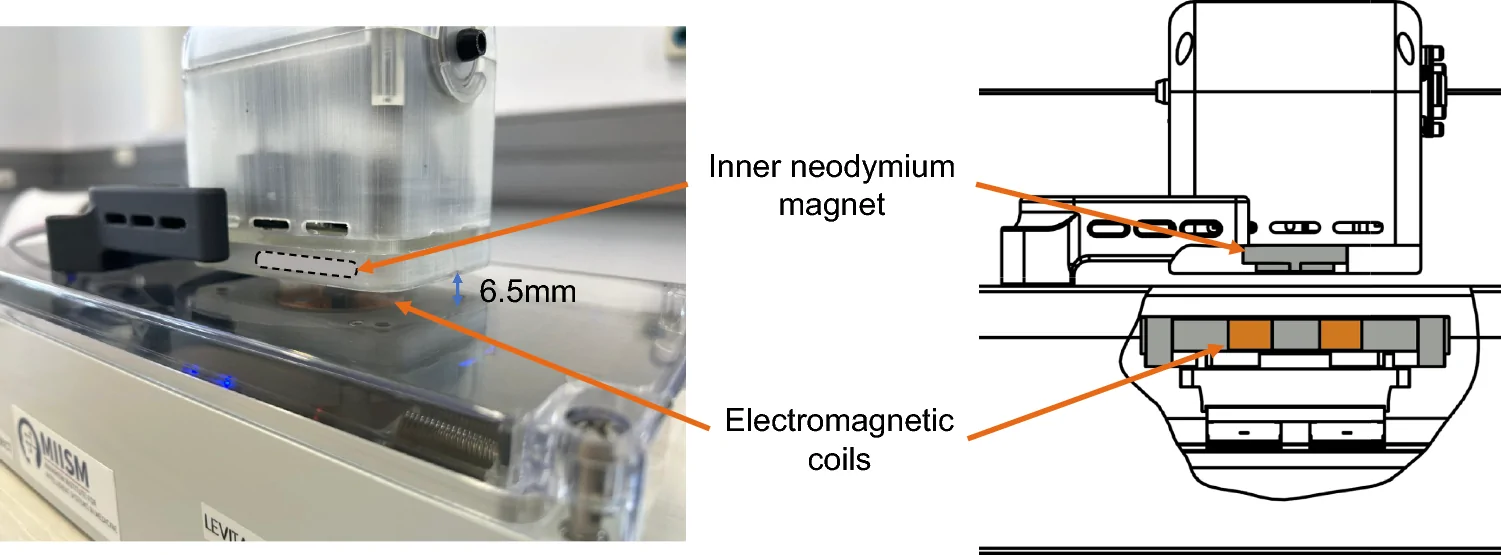
Levitating module over the electromagnetic coils during operation, with an approximate clearance of 6.5 mm. Arrows indicate the coils and the internal neodymium magnet

In the present prototype, the linear stroke of the dynamic module is approximately 170 mm. Initial vascular access is assumed to be performed manually, while the robot is intended for the local fine-control phase. An array of electromagnetic coils generates the lifting force for the dynamic module. The coil current is regulated to maintain the levitation height of a neodymium magnet embedded in the cartridge housing. The coil platform is mounted on a motorized linear guide, which provides the translational displacement required for catheter advancement. The coils and actuator are enclosed inside a sealed lower housing and remain isolated from the sterile field (Fig. 3).

### Sterile interface

The sterile-interface concept separates reusable robotic components from the catheter and guidewire. The reusable base and dynamic module can be enclosed in sterile covers, while the disposable cartridge is the only component in direct contact with the catheter. The cartridge is approximately 100 mm long, and the robot footprint is approximately 340 × 180 mm.

The sterility claim is design-based only. No microbiological sterility or contamination testing was performed. Therefore, this work should be interpreted as a functional validation of the sterile-interface concept, not as a sterility validation study. Dedicated validation aligned with relevant sterile-barrier standards, such as ISO 11607–1:2019 [9], will be required before clinical translation.

### Control software and hardware

Control follows a two-tier architecture. An Arduino microcontroller with a CNC shield runs the low-level loop at approximately 1 kHz and drives the stepper motors. A MATLAB layer provides the graphical interface and high-level motion commands through serial USB communication. The estimated end-to-end latency is 5–20 ms. The architecture is not hard real-time but was adequate for the present proof-of-concept experiments.

### Experimental setup

**Fig. 4:**
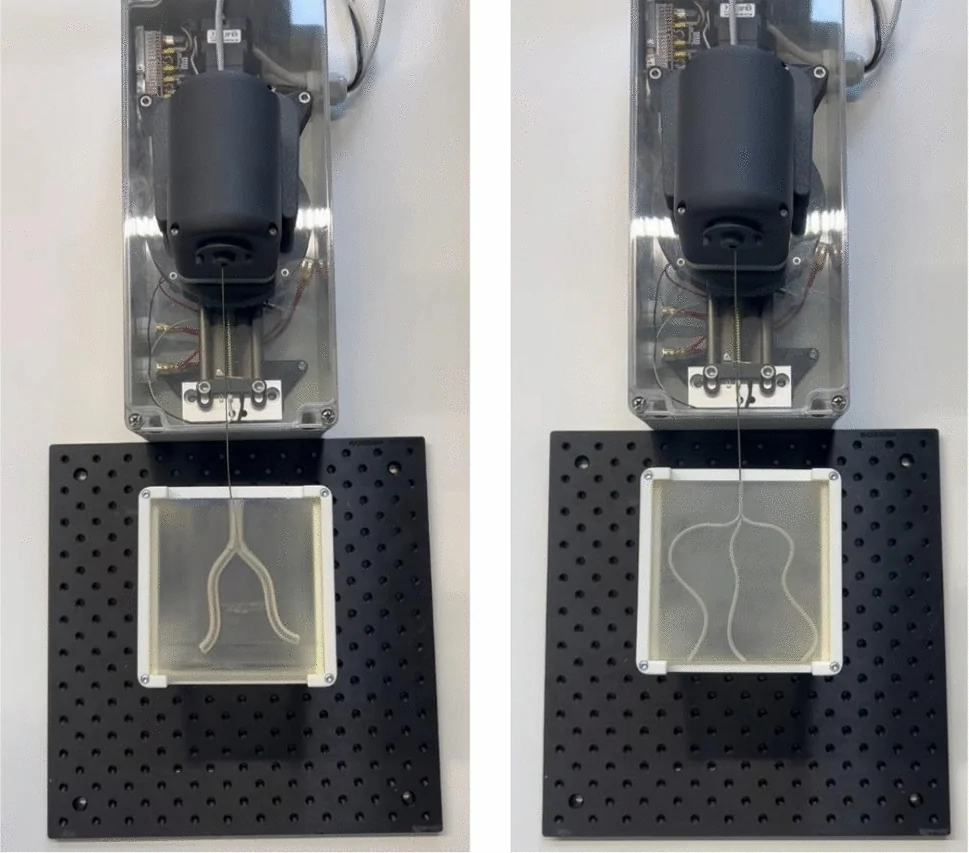
Vascular phantoms used for the experimental evaluation. The models reproduce bifurcated vessel geometries and were fabricated in transparent material to allow visual inspection during catheter navigation tests

The system was evaluated in transparent vascular phantoms reproducing bifurcated vessel geometries (Fig. 4). The channels had diameters of approximately 1–5 mm, and the transparent material allowed direct visual inspection during catheter and guidewire navigation.

The protocol included three functional test conditions, summarized in Table 1: catheter insertion–retraction cycles, combined guidewire–catheter cycles, and branch-selection trials. Success in insertion–retraction tests was defined as completion of the full programmed cycle without loss of grip. Success in combined guidewire–catheter tests was defined as advancement of the guidewire to the bifurcation followed by catheter advancement using the same device. Navigation stability was defined as first-attempt entry into the intended branch.

Commercial off-the-shelf 4F–8F catheters were tested, corresponding to outer diameters of approximately 1.3-−2.7 mm. The programmed advancement speed was 0.017 m/s. The device was operated by the first author and two uninvolved colleagues. Data were collected through visual observation and video tracking, which were sufficient for the discrete success/failure outcomes reported here but did not provide continuous force or position measurements.

## Results

The prototype demonstrated controlled catheter manipulation in the vascular phantoms. The functional outcomes and main experimental parameters are summarized in Table 1. The system completed 10/10 catheter insertion–retraction cycles without loss of grip. It also completed 10/10 combined guidewire–catheter cycles, in which the guidewire was advanced first and the guide catheter was subsequently inserted using the same device.

Branch-selection navigation was successful on the first attempt in 8/10 trials. The intended left branch was reached in 5/5 trials, and the intended right branch was reached in 3/5 trials. In successful trials, guidewire rotation allowed the operator to orient the guidewire tip toward the selected branch before advancement. The device maintained magnetic torque transmission during these operations.

The disposable cartridge could be mounted and removed without difficulty during bench operation. Magnetic coupling was established immediately when the external drive pulley was aligned with the cartridge magnets. Commercial 4F–8F catheters were manipulated without changing the hardware configuration.

**Table 1:** Summary of the experimental evaluation in vascular phantoms

Item	Value	Description
*Functional tests*		
Catheter insertion–retraction	10/10 cycles	Full advance and retract along the linear stroke; success defined as completed cycle without loss of grip
Combined guidewire + catheter	10/10 cycles	Guidewire advanced first to the bifurcation, followed by guide catheter insertion using the same device
Branch-selection navigation	8/10 trials	First-attempt entry into the intended branch: 5/5 left and 3/5 right
*Experimental parameters*		
Programmed advancement speed	0.017 m/s	Constant programmed speed used during the bench experiments
Catheter size range tested	4F–8F	Off-the-shelf catheters with outer diameters of approximately 1.3-−2.7 mm

**Table 2:** Comparison with representative robotic catheter platforms

System	Actuation	Sterile interface	Main limitation
CorPath GRX [10]	Cassette-based robotic drive	Single-use cassette	Dedicated platform and proprietary workflow
Chen et al. [11]	Cable-driven distal catheter	Not reported	Requires purpose-built catheter
Bao et al. [12]	Cooperation-based graspers	Not reported	Mechanically complex architecture
Kantu et al. [13]	Lead-screw and friction-roller drive	Not reported	Contact-based drive with slippage risk
Magnetic navigation [14]	External magnetic field	Platform-dependent	Dedicated infrastructure and instruments
FlyCaD	Magnetic coupling across sealed wall	Disposable cartridge	Early-stage prototype

At the programmed advancement speed of 0.017 m/s, the 5–20 ms control-chain latency corresponds to a positional delay below 0.4 mm. This value is smaller than the 1–5 mm phantom channel diameter. Therefore, positional uncertainty in the present prototype was dominated by mechanical factors rather than command latency.

The levitation mechanism enabled contactless operation during proof-of-concept testing but revealed a relevant limitation. Above a certain axial load, the dynamic module lost stability and dropped out of the electromagnetic field. This threshold was not measured with instrumented equipment. This observation identifies levitation stability as one of the main subsystems requiring further development.

## Discussion

This work presents a proof-of-concept robotic catheter-drive architecture that combines contactless magnetic torque transmission with a disposable sterile cartridge. The principal novelty is the transmission of actuation across a sealed sterile wall while keeping the catheter-contacting component simple and replaceable. The results show that this concept can provide sufficient functional performance for basic catheter advancement, guidewire rotation, and branch selection in transparent bifurcated vascular phantoms.

Commercial robotic systems, such as the CorPath GRX (Corindus Inc., Waltham, USA), have demonstrated clinical maturity using sterile disposable interfaces. Similar to these systems, FlyCaD addresses sterility management at the design level. However, the proposed architecture reduces mechanical contact pathways across the sterile barrier and aims to simplify the disposable interface [10]. In contrast to platforms that rely on dedicated cassettes or proprietary instrument workflows, FlyCaD was designed to remain compatible with commercial catheters within the tested 4F–8F range.

Mechanical catheter-driving systems based on rollers, grippers, or friction mechanisms can achieve effective linear motion but may require complex disposable interfaces and can be sensitive to slippage. Purely magnetic approaches offer miniaturization and remote actuation but often require dedicated instruments or costly magnetic-navigation infrastructure [5, 11–14]. FlyCaD takes a hybrid direction: magnetic coupling is used locally to transmit torque across a sterile barrier, while the catheter itself remains a standard clinical instrument.

Table 2 compares FlyCaD with representative robotic catheter platforms.

Several limitations must be considered. The evaluation was limited to transparent phantoms and did not reproduce full clinical frictional, anatomical, hemodynamic, or imaging conditions. Metrics were based on visual observation and video tracking; continuous force, torque, and position measurements were not collected. The levitation subsystem and sterility concept also require instrumented and microbiological validation.

The levitation module is the least mature subsystem. It enabled contactless translation but withstood only a limited axial load before losing confinement. We are therefore exploring a magnetic-attraction mechanism with a mechanical separator that preserves the sterile barrier while improving robustness under axial and non-axial loading. Future work will address force and position characterization, improved linear actuation, wireless operation, sterility validation, and real-time control.

## Conclusion

This work demonstrated FlyCaD, a robotic catheter-drive system that combines contactless magnetic torque transmission with a disposable sterile cartridge. The prototype enabled catheter advancement, retraction, and guidewire rotation in bifurcated vascular phantoms, completing 10/10 catheter insertion–retraction cycles, 10/10 combined guidewire–catheter cycles, and 8/10 first-attempt branch-selection trials.

The system should be interpreted as an early-stage functional demonstrator rather than a clinically validated robotic platform. Future work will focus on instrumented force and position characterization, improved linear actuation, wireless operation, sterility validation, and real-time control.

## Supplementary information

A supplementary video is provided showing the preparation of the device and its operation during preliminary tests. The material illustrates the assembly of the disposable cartridge, the magnetic coupling procedure, and the achieved catheter manipulation functions.

## Supplementary Information

Below is the link to the electronic supplementary material.

Supplementary file 1 (mp4 269547 KB)
